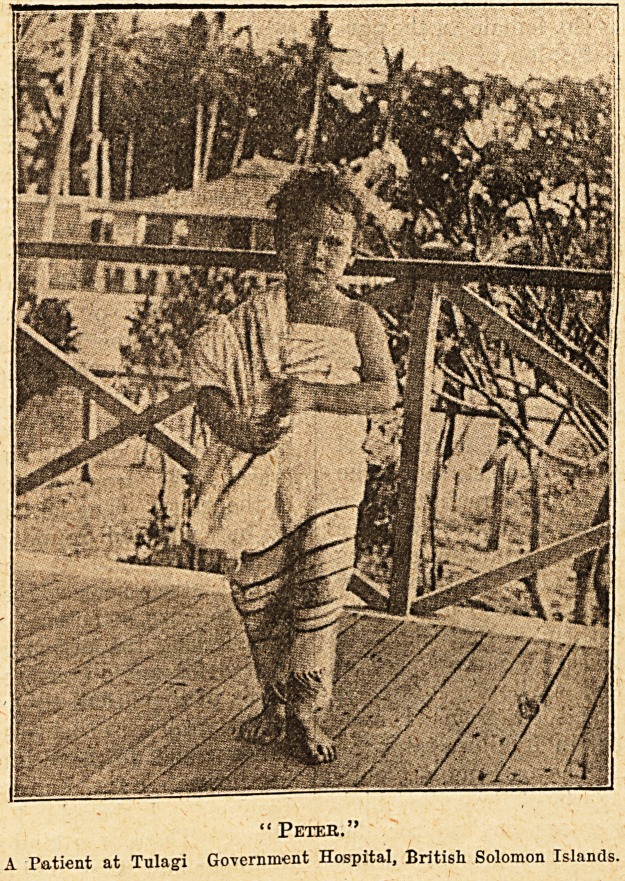# Round the Hospitals

**Published:** 1917-08-18

**Authors:** 


### ROUND THE HOSPITALS.

Eighteen years ago Miss Tinniswood entered the Dewsbury and District Infirmary as a probationer, where she worked for three years, and then received her certificate. She subsequently became a charge nurse, and rose to the rank of senior nursing sister. In October 1908—*i.e.*, nine years ago, Miss Tinniswood was unanimously elected matron of the institution, in succession to the late Miss Grime. Dewsbury held Miss Tinniswood in high esteem on account of her ability, devotion to her profession, and the possession of a lovable character, with an unmistakable charm of manner. Her eighteen years' service won for her the love of her staff, and the warm appreciation and confidence of the patients and of the inhabitants of Dewsbury. Two years ago she had a serious breakdown; an affection of the heart was diagnosed, and, to the regret of all who knew her, she died on the 6th inst. at the infirmary. Her funeral will be memorable in the history of Dewsbury on account of the large and representative attendance, coupled with the fact that the coffin was borne from the infirmary to the bier and into the parish church by members of the honorary medical staff, and later to the grave by members of the infirmary board. The board of management testify that Miss Tinniswood was throughout a most kind, able, and faithful servant of the institution. The Ladies' House Committee have passed the following resolution: "This meeting of the Ladies' House Committee deeply regrets the death of our esteemed matron, Miss Tinniswood, who was a devoted worker for the institution, and ever had the care of the patients in her mind." We know by life-long experience that a good matron is the most knowledgeable and indispensable of hospital officers. We give this full account of the proceedings at Miss Tinniswood's funeral because they demonstrate a splendid spirit of unity and personal service in work for the sick, with that just appreciation of a faithful officer whose loss will be long felt by all connected with her hospital. These encouraging incidents in the working of a voluntary hospital point the true way to life, and happiness in institutional life.

Miss Emma Maud McCarthy, R.R.C., has been promoted to the rank of Matron-in-Chief, Queen Alexandra's Imperial Military Nursing Service. Miss McCarthy has been temporary Matron-in-Chief in France since the beginning of the war, and her long and continuous work brought on an illness which laid her up for a time. It will be remembered that she was visited by Queen Mary during her recent visit with His Majesty the King to the Front, and that she came back to England with Her Majesty. Miss McCarthy was received at Marlborough House by Queen Alexandra on the 9th inst., and has since returned to take up her duties, with added responsibilities, across the water. Few who have not experienced the strain can have anything like a full idea of the wear and tear of a high office in connection with the war for a lengthened period. Those of us whose duty has lain at home who are in a similar position in regard to responsibility and work know full well what it means, however, and in congratulating Miss McCarthy on her well-earned promotion we can state with authority that she is entitled to receive the grateful acknowledgments of all classes in Great Britain.

The question of registration is at the moment in an interesting position, from the fact that on the initiative of Dr. Chapple, M.P., and a letter written by the Hon. Arthur Stanley, C.B., M.P., Chairman of the Council of the College of Nursing, Limited, negotiations between the College and the Central Committee are open to be resumed, at the request of the latter Committee, with a view to secure an agreed Bill. Despite the fact here stated, however, no such request has been made by the Central Committee, who will weaken their position to the point of failure if the delay continues much longer. It is a thousand pities, as we have pointed out more than once, that the small and prejudiced few sowers of disunion should be, as Mr. Stanley is afraid, "working rather to serve their own selfish ends, and, while they were agreeing to negotiate, were openly and secretly doing their absolute best to damage the people with whom they pretend to be working." The British nursing profession are fully aware of this fact, and unless the opportunity to-day open to the Central Committee to secure an agreed Bill is embraced, the Central Committee may speedily perish from attrition.

---

ONE of the most useful and unobtrusive of the Empire's organisations for nursing is the Colonial Nursing Association, Imperial Institute, S.W. 7. The twenty-first report, for the current year, is just to hand, and is well illustrated by excellent illustrations of the small-pox wards of Singapore, an ambulance train in British East Africa, and "Peter," a patient at Tulagi Government Hospital, British Solomon Islands, which latter we reproduce. Like most other Nursing Associations, the Colonial, since the outbreak of war, has experienced difficulties in filling many vacancies. These the enterprise of the management has successfully overcome, and no decrease in the number of Government nurses sent out has occurred, whilst the number of private nurses has been increased by two. The numbers would have been larger had it not been for the submarine activities, which caused the Foreign Office to impose restrictions which prevented women from leaving the country. As a result, several nurses, though appointed to Government posts, were unable to embark, and the passages of a few private nurses had to be cancelled. Such is wartime! But the detained nurses are, meanwhile, working temporarily in military hospitals at home. Three hundred and one nurses have been employed abroad, of whom seventy have been working as private nurses, 190 in Government service in the Crown Colonies, seventeen have been employed by the Government of Western Australia, eight by the Union of South Africa, and sixteen in other hospitals not under Government. It is interesting to note that four Colonial Nursing Association nurses on the staff of the Ceylon and Federated Malay States Government Hospitals have been promoted to matronships in those Colonies. One nurse has been sent out to Bangkok, Ceylon, Japan, Lisbon, Mauritius, Oporto, and Singapore respectively; while six nurses have been sent to Shanghai, and four to South Africa. The report includes that of the Scottish branch for the year ending March 31, 1917, a list of the nurses employed on which has its own importance, and should be remembered for reference, the audited balance-sheet and accounts, which, we are glad to see, show a small excess of income over expenditure for the year, and a map showing the posts filled by the Colonial Nursing Association nurses, distinguishing those employed in Government and non-Government hospitals from those in private service. The fruit of someone's happy idea will be found opposite page 40, where a Roll of Merit appears, containing the names of nurses who have retired from the Association after long and full service; and we are glad to see that the length of that service is in each case duly noted. Nurse Ann L. F. Milligan appears to have served the Association faithfully for the greatest number of years (eighteen), though several others have given notably long service out of the seventeen names entered so far on the Roll.

"PETER."
A Patient at Tulagi Government Hospital, British Solomon Islands.

---

AN exhibition of wounded warriors' work was held recently in the Nurses' Garden of the Bristol Royal Infirmary, where instruction in various handicrafts is given by ladies to wounded soldier patients in the 2nd Southern General Hospital. The soldiers have been taught stencilling, basketmaking, fancy and other needlework, rug-making, drawing, and painting. Prizes were awarded to Sergeant Bridge, Corporals Osborne, Howes, McGill, A.S.C., and Meads, Gunner Sandford, Sapper Stockham, R.E., Bandsman Neeson, and Privates Wink, Lloyd, Archer, Lilly, Tongue, and Shawney.

---

WE are glad to note that Dr. Edwards defended the nurses at the Swansea Workhouse, who he properly insisted had been unfairly treated by being compelled to perform domestic duties in addition to their proper work, owing to the great shortage of female labour. The Guardians, in consequence, have taken steps to abolish the abuses in question, and to employ the requisite labour for domestic duties.

## Figures and Tables

**Figure f1:**